# Lipid-Lowering Efficacy of Kuding Tea in Patients With Metabolic Disorders: A Systematic Review and Meta-Analysis of Randomized Controlled Trials

**DOI:** 10.3389/fnut.2022.802687

**Published:** 2022-04-28

**Authors:** Zhonghui Jiang, Zhuqing Lu, Tianyi Wang, Yilian Wang, Jianfeng Chu, Keji Chen, Zhuye Gao

**Affiliations:** ^1^Department of Cardiology, Xiyuan Hospital, China Academy of Chinese Medical Sciences, Beijing, China; ^2^National Clinical Research Center for Chinese Medicine Cardiology, Beijing, China; ^3^Academy of Integrative Medicine, Fujian University of Traditional Chinese Medicine, Fuzhou, China; ^4^Fujian Key Laboratory of Integrative Medicine on Geriatrics, Fujian University of Traditional Chinese Medicine, Fuzhou, China

**Keywords:** kuding tea (KT), lipid profile, systematic review, meta-analysis, metabolic disorders

## Abstract

**Background:**

Kuding tea (KT), traditional tea material and widely used in China, has been found to have lipid-lowering effect in clinical and experimental studies. However, there has been no systematic review of the evidence on this subject.

**Methods:**

Eight electronic databases were searched from database inception until September 2021 for relevant randomized controlled trials (RCTs). We used the Cochrane Reviewer’s Handbook to assess the quality of the included studies. Weighted mean difference (WMD) and 95% confidence interval (CI) were used to measure the pooled effect size by random-effects model. Funnel plot, Egger regression test, and the Begg’s test was used to assess publication bias.

**Results:**

Eight RCTs involving 716 patients were included in our meta-analysis. Comparing with the control group, KT group reduced serum total cholesterol (TC) levels (WMD: −0.56 mmol/L; 95% CI: −0.64, −0.47; *I^2^* = 56.56%; *P* = 0.00), triglyceride (TG) levels (WMD: −0.30 mmol/L; 95% CI: −0.35, −0.24; *I*^2^ = 88.60%; *P* = 0.00), and low-density lipoprotein cholesterol (LDL-C) levels (WMD: −0.29 mmol/L; 95% CI: −0.37, −0.21; *I^2^* = 89.43%; *P* = 0.00), but no significant effects on high-density lipoprotein cholesterol (HDL-C) (WMD: 0.07 mmol/L; 95% CI: −0.02, 0.16; *I*^2^ = 93.92%; *P* = 0.12). The results of sensitivity analysis were not altered after removing each study in turn. Subgroup analyses showed that KT intervention period was the source of heterogeneity. Following analysis, results revealed that long-term (>4 weeks and ≤8 weeks) use of KT increased HDL-C levels (WMD: 0.19; 95% CI: 0.13, 0.25). In addition, both the sensitivity analysis and subgroup analysis showed that our results were robust. No potentially significant publication bias was found from the funnel plot, Begg-Mazumdar correlation test and Egger regression test.

**Conclusion:**

KT supplementation can effectively improve lipid profile and KT is a promising approach to reduce blood lipid level in patients with metabolic disorders.

**Systematic Review Registration:**

[www.crd.york.ac.uk/prospero], identifier [CRD42020221850].

## Introduction

Metabolic disorders are multiple risk factor for cardiovascular disease (CVD), including dyslipidemia, obesity (particularly central adiposity), insulin resistance and elevated blood pressure (BP) (1, 2). It is estimated that more than 1 billion people worldwide are affected with metabolic disorders (2), and patients with metabolic disorders are twice as susceptible to develop CVD in the next 5–10 years as non-metabolic disorders patients (3). Clinical trials of therapies lowering lipids [e.g., Low-density lipoprotein cholesterol (LDL-C) and triglyceride (TG)] have consistently shown to reduce the risk of cardiovascular (CV) events (4–9). Lifestyle modifications (such as exercise and diet) can help patients preclude or reduce total cholesterol (TC), high-density lipoprotein cholesterol (HDL-C), LDL-C, and TG levels (6, 9). The use of alternative therapies, especially natural medicines and dietary supplements, in the treatment of metabolic disorders has rapidly increased and received increasing attention worldwide over the recent decade (10, 11).

Kuding tea (KT) has a total of 12 species belonging to six families and six genera (12, 13), is a traditional Chinese herb beverage commonly consumed in China, which has been reported to have many health benefits, such as anti-obesity (14), anti-inflammatory (15), anti-oxidation (16), modulating gut microbiota (17), and lowering lipid levels (18). Previous animal studies have shown that kudingcha dicaffeoylquinic acids (diCQAs) reduced the liver and adipose tissue mass, serum TC and LDL-C concentrations in high-fat diet fed mice (19); however, despite numerous randomized controlled trials (RCTs) on the effect of KT on lipid levels, as of yet no systematic review and meta-analysis of these evidence, so we conducted a systematic review and meta-analysis of RCTs involve the KT supplementation in the regulation of lipids.

## Methods

### Search Strategy

The meta-analysis was conducted according to the Preferred Reporting Items for Systematic Reviews and Meta-analyses (PRISMA) guidelines for RCTs (20) and registered with International Prospective Register of Systematic Reviews (PROSPERO) (registration number: CRD42020221850).^1^ We searched the PubMed, EMBASE, MEDLINE, Cochrane Library, the Cochrane Central Register of Controlled Trials, China National Knowledge Infrastructure Database (CNKI), Wanfang, and VIP from the inception dates to September 2021 for RCTs, without language restrictions. Two reviewers screened through the above electronic databases. Supplementary Appendix A shows the detailed search strategies. We obtained additional articles of research expertise in lipids from domain expert, and searched for additional studies from a list of references, related studies, and reviews of studies identified in a literature search.

### Selection Criteria

We screened the selected studies in strict accordance with the principles of PICOS (Participants, Interventions, Comparisons, Outcomes, and Study design).

Participants: Patients with metabolic disorders were diagnosed according to recognized diagnostic criteria (IFD, WHO, or NCEP-ATP III) (3).

Interventions: Supplement KT or related supplements. The species name and distribution of KT are shown in Table 1 (12, 13).

**TABLE 1:** Species name and distribution of kuding tea (KT).

Species name	Distribution
*Ilex Kudingcha C.J. Tseng*	Guangxi, China; Hainan, China
*Ilex latifolia* Thunb	Zhejiang, China; Hunan, China
*I.cornuta* Lindl	Zhejiang, China; Jiangsu, China
*Ligustrum pedunculare* Rehd.	Sichuan, China
*L.japonicium* Thunb.var.pubscens	Guizhou, China
*L.purpurascens* Y.C.Yang	Liaoning, China; Hebei, China
*L.henryl* Hemsl.	Sichuan, China; Yunnan, China
*L.lucidum* Ait.	Sichuan, China
Osmanthus matsumuranus Hayata	Guangxi, China; Hainan, China
Cratorylum prumflorum Dyer	Yunnan, China; Guangxi, China
*E.corylifolia* C.Hwright	Guizhou, China; Yunnan, China
*Itea ilicifolia* Oliv.	Guizhou, China

Comparison: No use of KT or KT-related supplements categories of exposure.

Outcome: Lipid parameters (TC, TG, HDL-C and LDL-C).

Study design: Randomized controlled trials (RCTs).

### Data Extraction and Quality Assessment

Two reviewers independently extracted the following information from each study using a standardized electronic form: first author, publication year, country, sample characteristics, study design, dose of KT, duration, baseline serum TC, TG, HDL-C, LDL-C concentration and other information including age and sex. Disagreements were resolved by consulting with a third investigator.

We used the Cochrane Collaboration risk of Bias Tool to assess the quality of the included studies in the following seven domains: the generation of random sequences, allocation concealment, blinded method used, integrity of the outcome data, selective outcome reporting, and other bias. Based on information provided by journal publications, we rated the risk of bias for each criterion as low, high, or unclear.

### Data Synthesis and Analysis

For statistical analysis, the SD in TC, TG, HDL-C and LDL-C from baseline was used to calculate the mean difference (MD) between the KT group and the control group. The pooled effect size was measured with weighted mean difference (WMD) and 95% confidence interval (CI). Statistical heterogeneity across studies was examined using Cochran’s Q-test (test level α = 0.1) and *I*^2^ statistic. The effects of binary and continuous risk factors were estimated using a random-effects model (DerSimonian and Laird method). Sensitivity analysis was conducted to determine the impact of each study on the overall effect size by using leave-one-out method, and subgroup analysis was performed to investigate potential sources of heterogeneity in the trials. Publication bias was assessed by funnel plot, Egger regression test, and the Begg-Mazumdar correlation test. Analyses were performed using Stata (version 16.0) and Review Manager (version 5.3).

## Results

### Search Results and Study Inclusion

**FIGURE 1:**
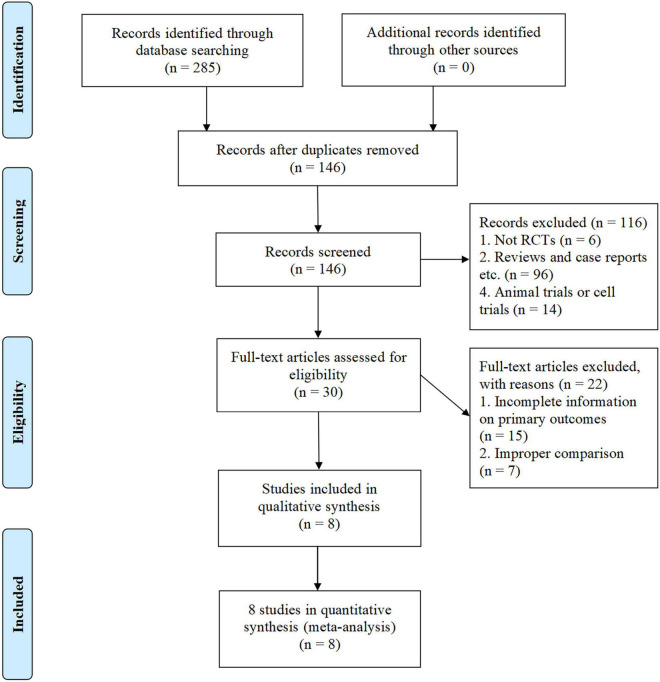
PRISMA flow diagram of study selection process.

The initial search from PubMed, EMBASE, MEDLINE, Cochrane Library, the Cochrane Central Register of Controlled Trials, CNKI, Wanfang, and VIP databases identified 285 potentially relevant articles for screening, of which 139 were removed due to duplication. The remaining 146 articles were further screened and 116 studies were excluded for the following reasons: study not designed as the RCTs (*n* = 6); experimental studies (*n* = 14); reviews and case reports etc. (*n* = 96). The full text of the remaining 30 articles was further screened, and 22 of them were excluded for the following reasons: incomplete information on primary outcomes (*n* = 15) and improper comparison (*n* = 7). Finally, 8 articles entered into our meta-analysis, involving 716 patients (361 [50.4%] in the KT group, 355 [49.6%] in the control group) (Figure 1). All included studies were conducted and published in China (21–28). The average sample size of these trials was about 90 participants (54 to 120 participants per trial). The course of treatment fluctuated between 2 and 8 weeks. The control group of all included studies were conventional western medicine, including atorvastatin (21, 28), simvastatin (23, 24), enalapril (22), perindopril (25), metformin hydrochloride tablets (26, 27). The characteristics of the included studies are shown in Table 2.

**TABLE 2:** Characteristics of included studies.

Included Trials	Country	Sample size		Population	Age (years)		Intervention		Duration (weeks)	Outcome
		IG	CG		IG	CG	KT dose (g/d)	Control group		
Li (21)	China	40	40	hyperlipidemia	67.4 ± 1.2	66.5 ± 1.6	150	atorvastatin	2	TC, TG, HDL-C, LDL-C
Liu et al. (22)	China	40	40	senile systolic hypertension	60–78	60–78	30	enalapril	4	TC, TG
Luo et al. (23)	China	60	55	hyperlipidemia	54.5 ± 6.4	56.5 ± 7.9	0.684	simvastatin	4	TC, TG, HDL-C, LDL-C
Luo et al. (24)	China	60	60	hyperlipidemia	40–70	40–70	0.684	simvastatin	4	TC, TG, HDL-C, LDL-C
Tao et al. (25)	China	43	44	menopausal women with hypertension	48.03 ± 3.77	47.55 ± 5.36	60	perindopril	8	TC, TG, HDL-C, LDL-C
Wang (26)	China	34	34	metabolic syndrome	47.8 ± 6.3	47.1 ± 5.9	20	metformin hydrochloride tablets	8	TC, TG, HDL-C
Yu and Zhao (27)	China	28	26	type 2 diabetes mellitus	46.7 ± 5.9	47.1 ± 5.8	20	metformin hydrochloride tablets	4	TC, TG, HDL-C
Zhao and Guo (28)	China	56	56	hyperlipidemia	57.3 ± 9.2	55.8 ± 10.1	18	atorvastatin	8	TC, TG, HDL-C, LDL-C

*IG, intervention group; CG, control group; KT, Kuding tea; TC, total cholesterol; TG, triglyceride; HDL-C, high-density lipoprotein-cholesterol; LDL-C, low-density lipoprotein-cholesterol.*

### Quality Assessment

**FIGURE 2:**
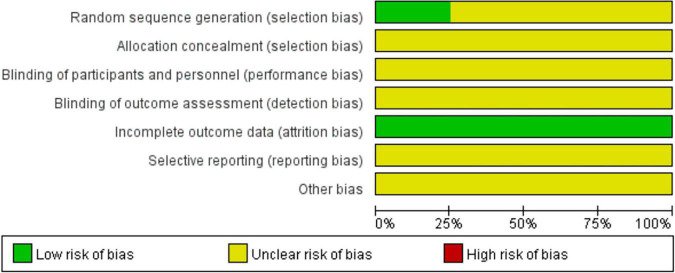
Risk of bias graph for included studies.

The quality assessment of the included studies was shown in Figure 2. All of the included studies (21–28) described the random sequence generation methods, among which 2 studies (25, 28) used computer-generated list of random numbers. The rest included studies only mentioned random, did not report it in detail. In addition, selective reporting, allocation concealment and blind were not very clear.

### Effects of Kuding Tea on Lipid Levels

#### Total Cholesterol

**FIGURE 3:**
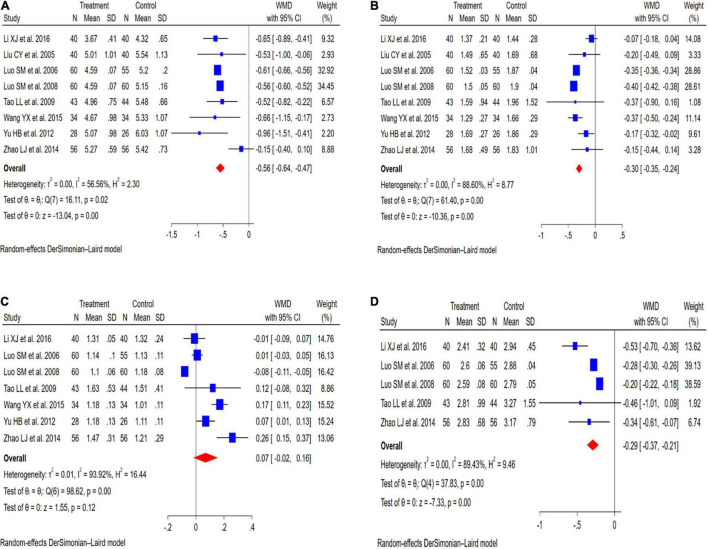
Meta-analysis results of KT supplementation for lipid levels. **(A)** TC levels. **(B)** TG levels. **(C)** HDL-C levels. **(D)** LDL-C levels.

**FIGURE 4:**
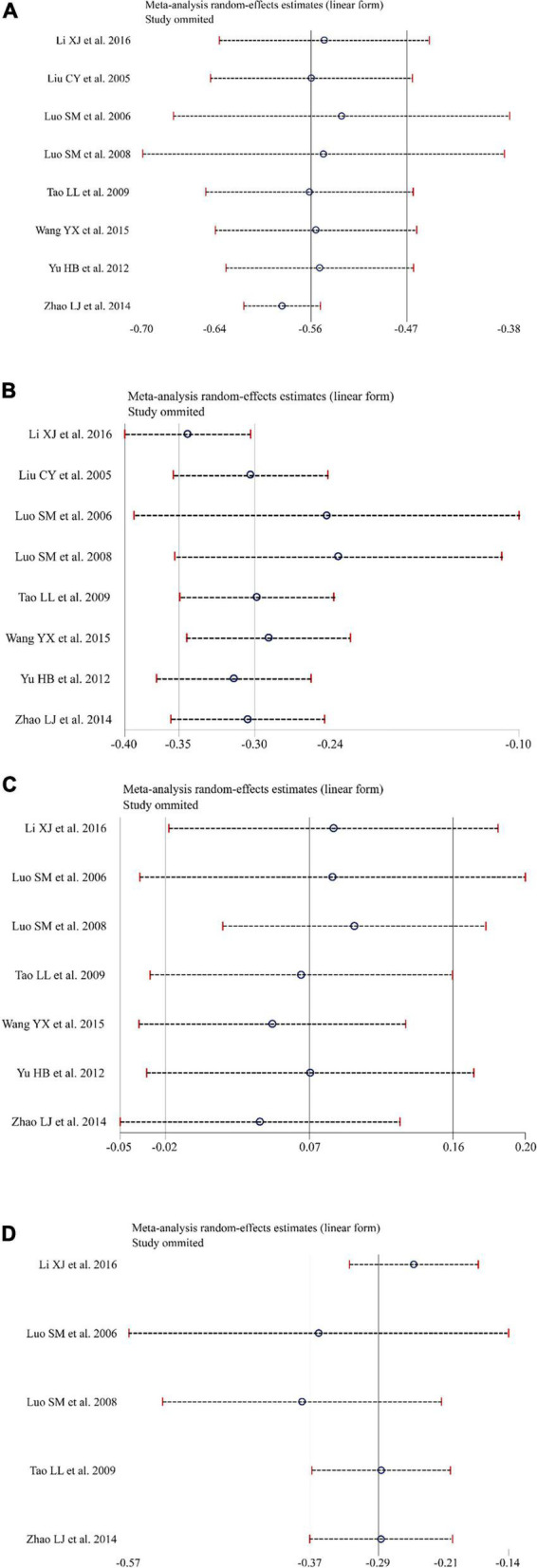
Sensitivity analysis was conducted to determine the impact of each study on the overall effect size by using removing each study in turn. **(A)** Sensitivity analysis for total cholesterol (TC). **(B)** Sensitivity analysis for triglyceride (TG). **(C)** Sensitivity analysis for high-density lipoprotein cholesterol (HDL-C). **(D)** Sensitivity analysis for low-density lipoprotein cholesterol (LDL-C).

**FIGURE 5:**
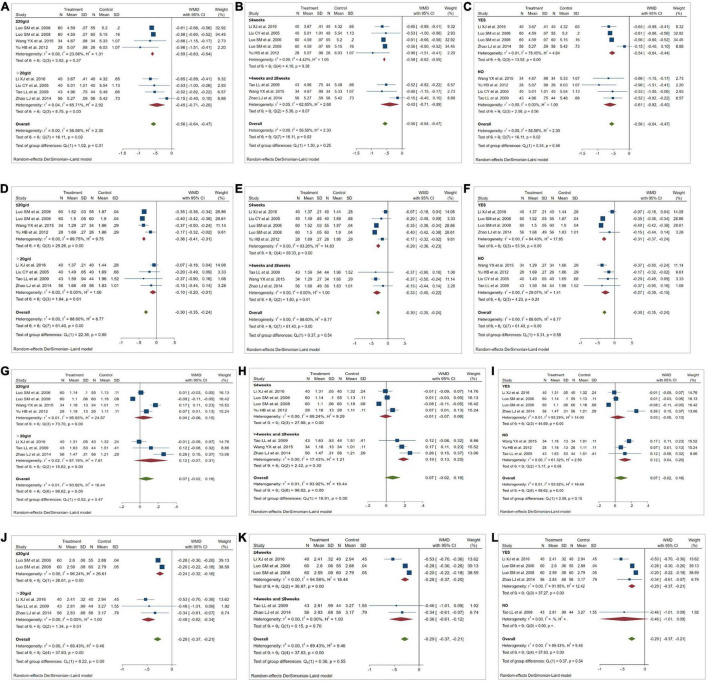
Results of subgroup analysis. **(A,D,G,J)** Effect of dose of Kuding tea (KT) on total cholesterol (TC), triglyceride (TG), high-density lipoprotein cholesterol (HDL-C). low-density lipoprotein cholesterol (LDL-C). **(B,E,H,K)** Effect of duration of KT intervention on TC, TG, HDL-C, LDL-C. **(C,F,I,L)** Effect of KT use and whether statins were used in the control group on TC, TG, HDL-C, LDL-C. WMD, weighted mean difference.

Eight studies (21–28) reported the effect of KT supplementation on TC levels (Figure 3A). Compared with the control group, KT group reduced TC levels (WMD: −0.56 mmol/L; 95% CI: −0.64, −0.47; *I^2^* = 56.56%; *P* = 0.00). Sensitivity analysis showed that the results did not change before and after sensitivity analysis (Figure 4A). Inter-group heterogeneity changed after subgroup analysis based on dose and duration of KT supplementation, and statins/non-statins as control group, but there was no significant difference in TC levels before and after subgroup analysis (Figures 5A–C).

#### Triglyceride

Eight studies (21–28) evaluated the effect of KT supplementation on TG levels. Our meta-analysis revealed a significant reduction in TG levels following KT supplementation (WMD: −0.30 mmol/L; 95% CI: −0.35, −0.24; *I^2^* = 88.60%; *P* = 0.00) (Figure 3B). A sensitivity analysis was performed that separately excluded the individual trials, and the results remained unchanged (Figure 4B). The heterogeneity has changed between different intervention time groups, dose and statins/non-statins as control group, but there were no significant differences before and after subgroup analysis (Figures 5D–F).

#### High-Density Lipoprotein Cholesterol

Effect of KT supplementation on HDL-C levels was investigated in seven trials (21, 23–28). As shown in Figure 3C, there was no statistically significant reduction of KT supplementation on HDL-C levels (WMD: 0.07 mmol/L; 95% CI: −0.02, 0.16; *I*^2^ = 93.92%; *P* = 0.12). The results of sensitivity analysis were not altered after removing each study in turn (Figure 4C). Subgroup analysis suggested that a dose of KT and statins/non-statins as control group had no significant impact on between-study heterogeneity (Figures 5G,I). However, long-term (>4 weeks and ≤8 weeks) use of KT may increase HDL-C levels (WMD: 0.19; 95% CI: 0.13, 0.25) (Figure 5H).

#### Low-Density Lipoprotein Cholesterol

Forest plots demonstrating the effects of KT supplementation on lipids are shown in Figure 3D. Our meta-analysis of seven studies (21, 23–25, 28) showed that KT supplementation decreased LDL-C (WMD: −0.29 mmol/L; 95% CI: −0.37, −0.21; *I*^2^ = 89.43%; *P* = 0.00). Sensitivity analysis showed no significant change in the overall estimate of effect size after excluding the individual trials (Figure 4D). Subgroup analysis showed no significant differences within subgroups based on the dose of KT, duration of KT supplementation and statins/non-statins as control group on LDL-C levels (Figures 5J–L).

### Publication Bias

**FIGURE 6:**
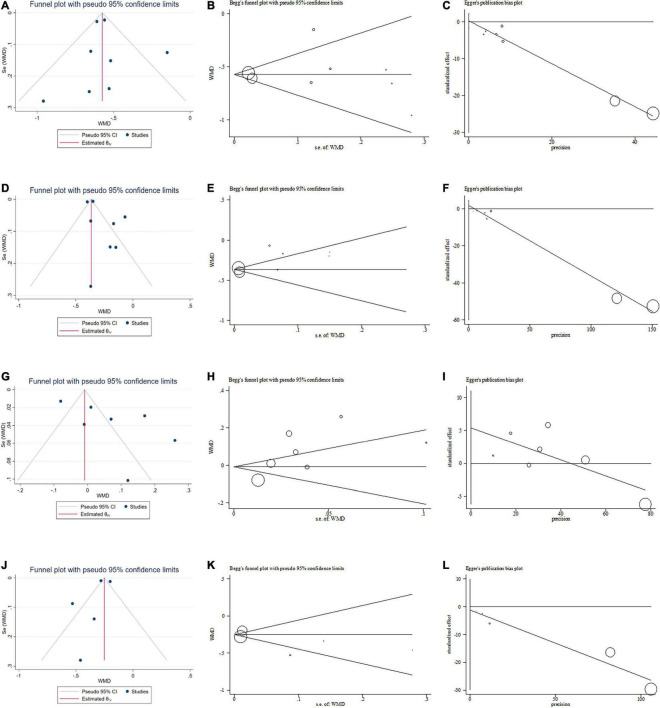
Publication bias. **(A)** Funnel plot for total cholesterol (TC). **(B)** Begg test for TC. **(C)** Egger test for TC. **(D)** Funnel plot for triglyceride (TG). **(E)** Begg test for TG. **(F)** Egger test for TG. **(G)** Funnel plot for high-density lipoprotein cholesterol (HDL-C). **(H)** Begg test for HDL-C. **(I)** Egger test for HDL-C. **(J)** Funnel plot for low-density lipoprotein cholesterol (LDL-C). **(K)** Begg test for LDL-C. **(L)** Egger test for LDL-C. WMD, weighted mean difference.

As shown in Figures 6A–L, funnel plot, Begg–Mazumdar correlation test and Egger regression test were used to evaluate the quality of meta-analysis, no publication bias for TC (Begg–Mazumdar correlation test, Kendall’s score = −6, continuity-corrected *z* = 0.62, continuity-corrected *P* = 0.54; Egger regression test, coefficient, 0.21; 95% CI, −1.73 to 2.14; *P* = 0.80), TG (Begg-Mazumdar correlation test, Kendall’s score = −4, continuity-corrected *z* = 0.37, continuity-corrected *P* = 0.71; Egger regression test, coefficient, 1.70; 95% CI, −1.34 to 4.73; *P* = 0.22), HDL-C (Begg–Mazumdar correlation test, Kendall’s score = 5, continuity-corrected *z* = 0.60, continuity-corrected *P* = 0.55; Egger regression test, coefficient, 5.41; 95% CI, −0.27 to 11.08; *P* = 0.06), and LDL-C (Begg–Mazumdar correlation test, Kendall’s score = 2, continuity-corrected *z* = 0.24, continuity-corrected *P* = 0.81; Egger regression test, coefficient, −1.24; 95% CI, −7.95 to 5.47; *P* = 0.60).

## Discussion

To our knowledge, this is the first meta-analysis to evaluate the effects of KT on lipid levels. This systematic analysis included eight RCTs involving 716 patients (361 in the KT group, 355 in the control group) of KT supplementation interventions for lipid levels and identified evidence of their beneficial effects on reducing serum TC, TG, and LDL-C levels. Subgroup analysis results showed that long-term (>4 weeks and ≤8 weeks) use of KT may increase HDL-C levels. In addition, both the sensitivity analysis and subgroup analysis showed that our results were robust.

Previous meta-analyses have shown that lowering TC levels, especially regimens that reduce LDL-C by about 1.5 mmol/L, not only reduce the incidence of ischemic heart disease (IHD), but also reduce by about a third the incidence of ischemic stroke, regardless of age, blood pressure, or or prerandomisation blood lipid concentrations (29, 30). In addition, studies have shown that the reduction of non-HDL-C by 1 mmol/L, the increase of HDL-C by 0.33 mmol/L and the decrease of total/HDL-C by 1.33 can reduce the mortality of IHD by about a third (30), while the decrease of TG by 0.1 mmol/L can reduce coronary events by 5% (31, 32). The weighted mean reductions in TC, TG and LDL-C appearing due to KT supplementation as observed in the present study (TC: 0.56 mmol/L; TG: 0.30 mmol/L; LDL-C: 0.29 mmol/L), may be important for the primary prevention of dyslipidemia. Moreover, KT has the advantages of low price (0.02–0.07 USD a day) and easy availability.

There are various mechanisms involved in the regulation of blood lipid levels by KT supplementation. It is suggested that KT extracts can modulate lipid metabolism disorders, upregulated mRNA expressions of peroxisome proliferator-activated receptor-α (PPAR-α), carnitine palmitoyltransferase-I (CPT-1), lipoprotein lipase (LPL), and cholesterol 7 alpha hydroxylase (CYP7A1) and downregulated mRNA expressions of peroxisome proliferator-activated receptor-γ (PPAR-γ) and CCAAT/enhancer-binding protein-alpha (C/EBP-α), which reduced adipocyte differentiation and lipid accumulation and improved dyslipidemia (33–36). In accordance with our results, several animal studies have reported that KT or its bioactive compounds significantly improved plasma lipid parameters, including TC, TG, and LDL-C (37–39). Moreover, it has also been confirmed that KT-derived saponins increased the content of serum lysoglycerophospholipids (Lyso-GPLs) and thus have a positive effect on lipid metabolism (40).

This meta-analysis has several limitations. First, heterogeneity of included studies was the most important limitation of this meta-analysis. Sensitivity analysis and subgroup analysis were conducted to analyze the factors (dose, duration, etc.) that may lead to considerable heterogeneity and explore the sources of heterogeneity. Heterogeneity changed after analysis, but the overall results were robust. Second, since all the studies were conducted in China, we cannot demonstrate whether the results can be stable in other parts of the world, and we cannot clarify the impact of differences in race, region and socio-economic characteristics on the results. In addition, the quality of some RCTs is poor, for example, unclear allocation concealment and blind method, although we adopt strict literature quality evaluation criteria.

The current available evidence to support the widespread use of KT is still limited and deserves additional attention, and KT supplements should never replace statins or other pharmacological approaches to reducing blood lipid levels as an adjuvant treatment strategy to effectively improve dyslipidemia and reduce the risk of CVD, especially in patients with statin intolerance and low to medium grade of dyslipidemia.

## Conclusion

This meta-analysis showed that KT supplementation seemed to improve metabolic disorders, and KT supplementation could reduce TC, TG, and LDL-C levels. The study also showed that long-term (>4 weeks and ≤8 weeks) supplementation of KT may increase HDL-C levels. This potential benefit needs to be further assessed through more rigorous and large-scale RCTs. Furthermore, this study provides useful information for the effect of KT on lipids and provides a way for the application of new lipid control therapies.

## Data Availability Statement

The original contributions presented in the study are included in the article/Supplementary Material, further inquiries can be directed to the corresponding authors.

## Author Contributions

ZJ designed the research and wrote the initial draft. ZJ, ZL, TW, and YW analyzed and synthesized the study data. JC, KC, and ZG managed and coordinated the responsibility for the research. All authors contributed to the article and approved the submitted version.

## Conflict of Interest

The authors declare that the research was conducted in the absence of any commercial or financial relationships that could be construed as a potential conflict of interest.

## Publisher’s Note

All claims expressed in this article are solely those of the authors and do not necessarily represent those of their affiliated organizations, or those of the publisher, the editors and the reviewers. Any product that may be evaluated in this article, or claim that may be made by its manufacturer, is not guaranteed or endorsed by the publisher.

## Footnotes

1
https://www.crd.york.ac.uk/prospero/
