# Supplementary material for: Lipid-Lowering Efficacy of Kuding Tea in Patients With Metabolic Disorders: A Systematic Review and Meta-Analysis of Randomized Controlled Trials
Source: Front Nutr. 2022 Apr 28;9:802687. doi: 10.3389/fnut.2022.802687 (PMC9096904; doi:10.3389/fnut.2022.802687)
Supplement: Supplementary file 1 [file Data_Sheet_1.docx]

**Appendix A. Search strategies**

| Database | Search | Search Strings |
| --- | --- | --- |
| PubMed | #1 | (((((((((Kuding cha) OR Ilex kudingcha) OR Ilex latifolia Thunb) OR Folium llicis Latifoliae) OR ilexlutifolia thumb) OR broadleaf holly leaf) OR Leaf of Chinese Holly) OR leaf of Broadleaf Holly) OR Ilex kudingcha C.J. Tseng) OR Kuding tea [All Fields] |
|  | #2 | ((((((((Total cholesterol) OR Epicholesterol) OR Cholesterin) OR Cholestenone) OR Cholesteryl ester transfer protein) OR Cholesteryl ester) OR Cholesterol ester) OR cholesterol) OR TC [All Fields] |
|  | #3 | ((((Triglycerides) OR Triacylglycerol) OR Triacylglycerols) OR Triglyceride) OR TG [All Fields] |
|  | #4 | ((((((((((((((((((High-density lipoprotein cholesterol) OR Lipoproteins, HDL) OR HDL Lipoproteins) OR High-Density Lipoprotein) OR Lipoprotein, High-Density) OR High-Density Lipoproteins) OR High Density Lipoproteins) OR Lipoproteins, High-Density) OR alpha-Lipoproteins) OR alpha Lipoproteins) OR Heavy Lipoproteins) OR Lipoproteins, Heavy) OR High Density Lipoprotein) OR Density Lipoprotein, High) OR Lipoprotein, High Density) OR alpha-Lipoprotein) OR alpha Lipoprotein) OR alpha-1 Lipoprotein) OR HDL-C [All Fields] |
|  | #5 | (((((((((Low-density lipoprotein cholesterol) OR Cholesterol, LDL) OR Low Density Lipoprotein Cholesterol) OR beta-Lipoprotein Cholesterol) OR Cholesterol, beta-Lipoprotein) OR beta Lipoprotein Cholesterol) OR LDL Cholesterol) OR Cholesteryl Linoleate, LDL) OR LDL Cholesteryl Linoleate) OR LDL-C [All Fields] |
|  | #6 | #2 OR #3 OR #4 OR #5 |
|  | #7 | randomized controlled trial[pt] |
|  | #8 | randomized clinical trial[pt] |
|  | #9 | controlled clinical trial[pt] |
|  | #10 | randomized[tiab] |
|  | #11 | placebo[tiab] |
|  | #12 | randomly[tiab] |
|  | #13 | trial[tiab] |
|  | #14 | groups[tiab] |
|  | #15 | #7 OR #8 OR #9 OR #10 OR #11 OR #12 OR #13 OR #14 |
|  | #16 | #1 AND #6 AND #15 |
| EMBASE | #1 | 'Kuding cha'/exp OR Kuding cha |
|  | #2 | 'Ilex latifolia Thunb'/exp OR Ilex latifolia Thunb |
|  | #3 | 'Kuding tea'/exp OR Kuding tea |
|  | #4 | #1 OR #2 OR #3 |
|  | #5 | 'Total cholesterol'/exp OR Total cholesterol |
|  | #6 | 'Triglycerides'/exp OR Triglycerides |
|  | #7 | 'High-density lipoprotein cholesterol'/exp OR High-density lipoprotein cholesterol |
|  | #8 | 'Low-density lipoprotein cholesterol'/exp OR Low-density lipoprotein cholesterol |
|  | #9 | #5 OR #6 OR #7 OR #8 |
|  | #10 | 'clinical trial' |
|  | #11 | #4 AND #9 AND #10 |
| Cochrane Library | #1 | MeSH descriptor: [Kuding cha] explode all trees |
|  | #2 | MeSH descriptor: [Kuding tea] explode all trees |
|  | #3 | (Ilex latifolia Thunb): ti,ab,kw |
|  | #4 | (Ilex kudingcha): ti,ab,kw |
|  | #5 | (Folium llicis Latifoliae): ti,ab,kw |
|  | #6 | (ilexlutifolia thumb): ti,ab,kw |
|  | #7 | (Leaf of Chinese Holly): ti,ab,kw |
|  | #8 | (broadleaf holly leaf): ti,ab,kw |
|  | #9 | (leaf of Broadleaf Holly): ti,ab,kw |
|  | #10 | (Ilex kudingcha C.J. Tseng): ti,ab,kw |
|  | #11 | #1 or #2 or #3 or #4 or #5 or #6 or #7 or #8 or #9 or #10 |
|  | #12 | MeSH descriptor: [Total cholesterol] explode all trees |
|  | #13 | MeSH descriptor: [Triglycerides] explode all trees |
|  | #14 | MeSH descriptor: [High-density lipoprotein cholesterol] explode all trees |
|  | #15 | MeSH descriptor: [Low-density lipoprotein cholesterol] explode all trees |
|  | #16 | #12 or #13 or #14 or #15 |
|  | #17 | #11 and #16 |
